# The relationship between Mediterranean-DASH diet intervention for the neurodegenerative delay (MIND) Diet and risk of breast Cancer: a case-control study among iranian adult women

**DOI:** 10.1186/s40795-022-00614-8

**Published:** 2022-10-27

**Authors:** Ebrahim Mokhtari, Sanaz Jamshidi, Hossein Farhadnejad, Farshad Teymoori, Bahram Rashidkhani, Parvin Mirmiran, Fahimeh Ramezani Tehrani, Zeinab Heidari

**Affiliations:** 1 Student Research Committee, Nutrition and Endocrine Research Center, Research Institute for Endocrine Sciences, Shahid Beheshti University of Medical Sciences, Tehran, Iran; 2 Nutrition and Endocrine Research Center, Research Institute for Endocrine Sciences, Shahid Beheshti University of Medical Sciences, Tehran, Iran; 3 Department of Community Nutrition, Faculty of Nutrition Sciences and Food Technology, National Nutrition and Food Technology Research Institute, Shahid Beheshti University of Medical Sciences, PO Box: 1981619573, Tehran, Iran; 4 Department of Nutrition, School of Public Health, Iran University of Medical Sciences, PO Box: 19395-4741, Tehran, Iran; 5 Reproductive Endocrinology Research Center, Research Institute for Endocrine Sciences, Shahid Beheshti University of Medical Sciences, Tehran, Iran; 6 Student Research Committee, Ahvaz Jundishapur University of Medical Sciences, Ahvaz, Iran

**Keywords:** MIND diet, Breast cancer, Dietary pattern, cancer, Women

## Abstract

**Background:**

choosing a healthier lifestyle and modifying dietary habits could prevent four million new people from developing cancer. Recently, a new index called the Mediterranean-dietary approach to stop hypertension (DASH) diet intervention for the neurodegenerative delay (MIND) diet has been developed. In the current study, we aimed to assess the relationship between the MIND diet and the risk of breast cancer (BC) among Tehranian adult women.

**Method:**

In this hospital-based, case-control study, 134 Tehranian women ≥ 30 years old with recently (< 6 months) diagnosed BC, confirmed histologically and 272 women of the same age as control were included. Dietary intakes were assessed in a personal interview using a valid and reliable semi-quantitative 168-item food frequency questionnaire. The odds ratio (OR) and 95% confidence intervals (CI) of breast cancer across tertiles of the MIND diet were determined using multivariable-adjusted logistic regression analysis.

**Results:**

In the crude model, participants in the highest tertiles had lower odds of BC [(OR = 0.57; 95% CI,0.34–0.95), P for trend = 0.020)] than those with the lowest scores on the MIND diet. After controlling for potential confounding variables, individuals in the highest tertile of the MIND diet had a 45% lower risk of BC [(OR = 0.55; 95% CI, 0.32–0.96), P for trend = 0.021)] compared with those in the lowest tertile. Also, in women with an abortion history, higher adherence to the MIND diet was associated with a lower risk of BC [(OR = 0.15; 95% CI, 0.04–0.52, P for trend = 0.002)].

**Conclusion:**

Our findings revealed that higher adherence to the MIND diet was associated with decreased BC risk, which was strongly observed among women with a history of abortion.

## Background

Breast cancer (BC) is the most prevalent cancer and is becoming the second cause of cancer mortality among women [1]. Besides unpreventable BC risk factors, including age, genetic mutations, premature menstruation, childbirth, later pregnancy, advanced menopause, dense breast tissue, hormone therapy, oral contraceptives, and cancer family history, the modifiable factors such as unhealthy diet and lifestyle, alcohol consumption, and smoking play a considerable role in the development of BC [2]. The World Cancer Research Centre stated that choosing a healthier lifestyle and modifying dietary habits could prevent four million newly diagnosed cancer patients from developing the disease [2].

Prospective studies have suggested that single nutrients or food groups are directly related to the risk of BC [3]. The association of pre-defined dietary patterns such as the alternative healthy eating index (AHEI), the Mediterranean diet (MED), and the dietary approach to stop hypertension (DASH) diet with BC has been investigated in previous studies. [3–8]. Studies linking the DASH and MED diets to the risk of BC have presented inconsistent results [6–9]. A meta-analysis of prospective studies shows that the DASH diet is associated with decreased BC risk factors [9]; however, some other studies do not confirm this finding [6]. Also, in the case of the MED, findings of a systematic review have suggested that higher adherence to the MED diet is not significantly associated with BC [8]; however, another study has found an inverse relationship between the MED and the risk of BC [7].

Recently, a new index called Mediterranean-DASH diet intervention for the neurodegenerative delay (MIND) diet has been developed using the related components of MED and DASH diet with neurodegenerative diseases [10]. The MIND diet has focused on higher consumption of natural plant-based foods and lower consumption of animal and high saturated fat foods; however, some changes have been applied. For example, in this index, scores are set only for the consumption of berries, and the rest of the fruits are omitted. Green leafy vegetables, other vegetables, seeds, berries, nuts, whole grains, fish, seafood, olive oil, and wine are among the healthy foods, and red meat, margarine and butter, cheese, sweets, fries, and fast food is unhealthy in this index [10–12].

Recently, several studies have shown the protective effects of this novel index on various neurodegenerative disorders such as Alzheimer’s [12, 13], depression [14], Parkinson’s [15], and Cognitive functions [16–19]. Although the MIND diet was initially developed for neurodegenerative diseases because it follows two dietary patterns, DASH and MED, it has been suggested that this healthy score may be a practical index for predicting the risk of other chronic diseases [10]. Aghamohammadi et al., in a case-control study, observed a 50% lower risk of BC among women who had higher adherence to the MIND diet [20]. Also, such a relationship was observed in glioma [21]. Besides, other studies have shown that a greater MIND diet score is related to a reduced risk of general obesity in Iranian adults [22].

The MIND diet is a novel pre-defined pattern that includes well-known and popular dietary indices, DASH and MED, in which only one study has investigated its association with the risk of BC. Regarding the high prevalence of BC in the Iranian population [23], the importance of examining possible effects of healthy dietary indices in reducing the risk of BC, and also the necessity of further assessment of MIND diet association with the risk of chronic disease in different study populations, we aimed to assess the MIND-BC relationship among Tehranian adult women.

## Materials and methods

### Study design and sample

In this hospital-based, case-control study, we recruited 136 women ≥ 30 years old with recently (< 6 months) diagnosed BC, confirmed histologically at Imam Hossain and Shohada hospitals, Tehran (Iran), between September 2015 and February 2016. The control group consisted of 272 women of the same age who were admitted to the same hospital for a broad spectrum of non-neoplastic diseases. Conditions among controls included traumas and orthopedic disorders, disk disorders, acute surgical conditions, and eye, nose, ear, or skin disorders.

Less than 8% of subjects approached for the interview refused to participate. Seven participants were excluded from the analysis because their reported energy intakes were outside the ± 3 standard deviation (SD) from the mean energy intakes of the population (five subjects in the control group and two subjects in the case group). Finally, 134 cases and 267 controls remained in the final analysis.

All participants filled out written informed consent. The ethics research committee approved the study’s protocol of the Student Research Committee, Shahid Beheshti University of Medical Sciences, Tehran, Iran (ethical approval code: IR.SBMU.RETECH.REC.1399.1281). In this study, the ethical standards of the Helsinki Declaration were followed in all procedures.

### Dietary assessment

Participants’ dietary intakes during the one year before diagnosis for cases or interview for controls were assessed in a personal interview using a valid and reliable semi-quantitative 168-item food frequency questionnaire (FFQ) [24]. Participants were asked to specify their daily, weekly, monthly, or yearly consumption frequency for each food item. Intakes were then converted to daily frequencies, and a manual for household measures was used to convert intake frequencies to daily grams of food intake [25]. The energy and nutrient content of foods was calculated using the USDA food composition table. For some traditional Iranian food items that are not included in the USDA database (e.g., traditional bread), the Iranian food composition Table  [26] was used. Due to Iranian cultural beliefs, alcohol consumption was not asked about and was unavailable for analysis.

### Calculation of MIND score

The MIND diet score was calculated based on the method introduced by Morris et al. [10]. MIND diet encompasses 15 dietary parameters, originally including ten components as brain-healthy food groups (green leafy vegetables, other vegetables, nuts, berries, beans, whole grains, fish, poultry, olive oil, and wine) and five components as brain unhealthy food groups (red meats, butter and margarine, cheese, pastries and sweets, and fast fried food). In the current study, we used 13 components to calculate the MIND diet score because the information on fast foods and wine consumption was unavailable in our dataset. First, participants were classified based on tertile categories of intakes of these 13 components. Participants in the lowest tertile of brain-healthy food groups intake were assigned a score of 0, those in the second tertile were assigned a score of 0.5, and those in the highest tertile were assigned a score of 1. For brain, unhealthy food groups, participants in the lowest, middle, and highest tertile were assigned a score of 1, 0.5, and 0, respectively. Finally, the overall MIND diet score was calculated by summing up all dietary component scores, and a score between 0 and 13 was given to each participant.

### Assessment of non-dietary exposures

Trained dietitians administered all other questionnaires and measurements during the same interview. Participants’ socio-demographic, lifestyle, and clinical information collected by general questionnaires, including age (years), age at menarche (years), age at first pregnancy (years), abortion history (yes, no), number of live births (number), breastfeeding history (month), menopausal status (pre-menopause, post-menopause), education (illiterate, less than a high school diploma, high school diploma and more), history of hormone replacement therapy (yes, no), oral contraceptive pills consumption history (month), benign breast diseases history (yes, no), cancer family history (yes, no), breast cancer family history (yes, no), bra wearing (day (yes, no), night (yes, no)), Marital status (single, married, divorced, widowed), smoking (yes, no), supplement intake (including calcium, iron, zinc, selenium, B complex, Vitamin C, folic acid, vitamin A vitamin C, β carotene, vitamin E, vitamin D, multivitamins-minerals, omega-3 fatty acids, and probiotics) (yes, no; If yes, the complementary information about dose and frequency), and anti-inflammatory drug use (yes, no). Also, data on physical activity levels were collected using a valid and reliable questionnaire [27].

Socio-economic-status (SES) was computed based on three components, including education levels (illiterate or low education vs. high education (diploma and upper degrees)), occupation status (jobless vs. having a part or full-time job or being retired), and the number of children (2 or lower vs. 3 or higher). Each variable was given a score of 0 or 1. Finally, SES was calculated by summing the score of the three components mentioned above for subjects. Participants with scores higher or equal to 2 and subjects with lower or equal to 1 were classified as high and low SES, respectively.

Weight was measured to the nearest 0.5 kg using a digital scale (Seca, Germany), with the participant wearing lightweight clothing and no shoes. Height was measured to the nearest 0.5 cm using by tape meter fixed to a wall. Body mass index (BMI) was calculated by dividing weight (kg) by the square of height (meter). Waist circumference (at the level midway between the lowest rib margin and the iliac) and hip circumference (at the widest point over the buttocks) were measured to the nearest 0.5 cm using a non-stretchable tape measure. Subsequently, the waist-hip ratio (WHR) was calculated.

### Statistical analysis

The normally distributed variables between the two groups were assessed using a histogram chart and Kolmogorov- Smirnoff test. The mean values of the two groups were compared using the student’s t-test, and the means of more than two groups were assessed using analysis of variance (ANOVA) for normally distributed variables. Also, non-parametric statistics, including the Mann-Whitney U test or the Kruskal-Wallis test, were used for variables without normal distribution. Moreover, categorical variables were compared using the chi-square test. The energy-adjusted values of the MIND diet using the residual method were calculated, and the MIND diet was categorized as tertiles based on the three equal categories among controls. The odds ratio and 95% confidence intervals (ORs and 95% CIs) of breast cancer across tertiles of the MIND diet were calculated using logistic regression analysis that adjusted for multiple covariates in different models. Different potentially confounders were adjusted in the final regression model, including age, age at first pregnancy, family history of cancer (yes, no), menopausal status (yes, no), anti-inflammatory drugs (yes, no), and vitamin D supplement (yes, no), energy, BMI, physical activity, smoking status (yes, no), and SES (high, low). After testing for interaction, analyses were stratified by abortion history (P-interaction < 0.05). Statistical tests were performed using SPSS software (v.16.0). Significance level was set at 0.05, and all P values were based on two-sided tests.

## Results

All participants’ mean ± SD of age and BMI were 47.9 ± 10.3 years and 29.4 ± 5.5 kg/m^2^, respectively.

The baseline characteristics of participants are presented in Table 1. Compared to control groups, individuals in the case group had higher age, first pregnancy age, post-menopause status percentage, and family cancer history (P < 0.05). In contrast, individuals in the case group had lower anti-inflammatory drug consumption, MIND diet score, and vitamin supplement intakes than the control group (P < 0.05). There were no significant differences between the two groups for other variables.

**Table 1:** Characteristics of breast cancer cases and controls, Iranian breast cancer case-control study, 2015–2016

Variables	Cases(n = 134)	Controls(n = 267)	P-value^†^
Age(yrs.): mean ± SD ^1^	49.5 ± 10.7	47.13 ± 10.1	0.03
Menarche age(yrs.): median (IQR)	14.0 (2.00)	13.0 (1.0)	0.77
Marriage age(yrs.): median (IQR)	19.0 (7.00)	18.0 (4.0)	0.07
First pregnancy age(yrs.): median (IQR)	20.0 (8.5)	19.0 (5.0)	0.04
BMI (kg/cm^2)^: median (IQR)	29.5(7.3)	28.5(7.7)	0.07
WHR: mean ± SD ^1^	0.9 ± 0.1	0.9 ± 0.1	0.61
Physical activity: median (IQR) (MET-h/day)	32.1 (6.2)	31.47 (6.1)	0.70
Smoking (Present smoker): n (%)	4(3.0)	9(3.4)	0.84
SES status (% high)	82 (63.1)	154 (57.9)	0.53
Breastfeeding time(*month*): median (IQR)	(53.0)36.0	44.0 (47.3)	0.13
Menopausal status: n (%)			0.04
pre-menopause post-menopause	62(46.3) 72(53.7)	153(57.3) 114(42.7)	
Marital status: n (%)			0.73
Single Married Divorced Widowed	9 (6.8%) 105 (78.9%) 5 (3.8%) 14 (10.5%)	16 (6.0%) 206 (77.4%) 13 (4.9%) 31 (11.7%)	
**Medical history**			
Breast cancer family history (YES): n (%)	11(8.2)	12(4.5)	0.134
Cancer family history (YES): n (%)	41(30.6)	55(20.7)	0.03
Benign breast diseases history (YES): n (%)	12(9.0)	14(5.3)	0.157
Inflammatory disease history (YES): n (%)	15(10.4)	35(13.2)	0.435
Abortion history (YES): n (%)	52(38.8)	78(29.2)	0.053
OCP use time(yrs.): median (IQR)	1.0 (5.0)	1.00 (5.00)	0.819
Anti-inflammatory drugs (YES): n (%)	10(7.5)	46(17.3)	0.01
HRT (YES): n (%)	7(5.2)	29(10.9)	0.061
**Dietary intakes**			
Daily energy intake(kcal/day): median (IQR)	2467 (890)	2549 (1068)	0.07
Carbohydrate (% of energy)	53.7 ± 6.6	54.2 ± 7.0	0.44
Protein (% of energy)	36.0 ± 6.7	35.1 ± 6.7	0.10
Fat (% of energy)	12.6 ± 2.0	13.0 ± 2.1	0.21
MIND score: mean ± SD ^1^	19.9 ± 5.0	21.0 ± 4.0	0.034
Vitamin D supplement (YES): n (%)	20(4.9)	65(24.4)	0.03
Calcium supplement (YES): n (%)	35 (26.1)	73 (27.3)	0.795
Iron supplement (YES): n (%)	20 (14.9)	45 (16.9)	0.621
Folic acid supplement (YES): n (%)	16 (11.9)	30 (11.2)	0.835
Omega-3 supplement (YES): n (%)	8 (6.0)	31 (11.6)	0.072
Herbal drug (YES): n (%)	26 (19.4)	72 (27.1)	0.093

^†^ Student t-test or Mann-Whitney was used for continuous variables, Chi-square test was used for categorical variables

^1^ Normal distribution

NS: non-significant

Table 2 shows the association between the MIND diet and the risk of BC. In the crude model, participants in the highest tertiles had lower odds of BC [(OR = 0.57; 95% CI: 0.34–0.95), P for trend = 0.020)] than those with the lowest scores for the MIND diet (> 22.62 compared with < 19.41). The association remained significant in the two following adjusted models and became slightly stronger. In the fully adjusted model, after controlling the confounding effect of age, age at first pregnancy, family history of cancer, menopausal status, anti-inflammatory drugs, vitamin D supplement, energy, BMI, physical activity, smoking status, and SES status, individuals in the highest tertile of MIND diet had 45% lower risk of BC [(OR = 0.55, 95% CI: 0.32–0.96), P for trend = 0.021)], compared with those in the lowest tertile.

**Table 2:** Odds ratio and 95% confidence intervals for the association between MIND and breast cancer, Iranian case-control study, 2015–2016

Variable	Tertiles of the MIND			
	I	II	III	P-trend
	< 19.41	19.41 to 22.62	> 22.62	
Median score	16.8	21.0	24.8	
cancer /control	65/88	32/89	36/88	
Crude	Ref (1.00)	0.48 (0.28–0.82)	0.57 (0.34–0.95)	0.020
Model 1^*^	Ref (1.00)	0.48 (0.28–0.82)	0.55 (0.33–0.93)	0.017
Model 2^†^	Ref (1.00)	0.44 (0.25–0.76)	0.55 (0.32–0.94)	0.018
Model 3^‡^	Ref (1.00)	0.41 (0.23–0.72)	0.55 (0.32–0.96)	0.021

^*^Model 1: adjusted for age and age at first pregnancy

^†^Model 2: adjusted for model 1 and family history of cancer (yes, no), menopausal status (yes, no), anti-inflammatory drugs (yes, no), and vitamin D supplement (yes, no)

^‡^Model 3: adjusted for model 2 and energy, BMI, physical activity, smoking status (yes, no), and SES (high, low)

The ORs for BC, across tertiles of the MIND diet between two groups with and without abortion history, are shown in Table 3. In participants with an abortion history, higher adherence to the MIND diet was associated with a lower risk of BC in crude models [(OR = 0.23, 95% CI: (0.08–0.62), P for trend = 0.002)]. In the full adjusted model, in participants with a history of abortion, those at the highest tertile of the MIND diet vs. those at the lowest tertile had 85% lower odds of BC [(OR = 0.15, 95% CI: 0.04–0.52), P for trend = 0.002)]. However, in participants with no abortion history, there was no significant association between the MIND diet and the risk of BC based on logistic regression analysis.

**Table 3:** Odds ratio and 95% confidence intervals for the association between MIND and breast cancer by Abortion history, Iranian case-control study, 2015–2016

Variable	Tertiles of the MIND			
	I	II	III	P-trend
**Abortion history**				
**Yes**				
MIND values	< 19.37	19.37 to 23.16	> 23.16	
Median score	17.0	21.0	25.0	
cancer /control	31/25	14/26	7/25	
Crude	Ref (1.00)	0.41 (0.17–0.97)	0.23 (0.08–0.62)	0.002
Model 1^*^	Ref (1.00)	0.44 (0.18–1.06)	0.19 (0.06–0.54)	0.001
Model 2^†^	Ref (1.00)	0.31 (0.12–0.83)	0.20 (0.06–0.58)	0.002
Model 3^‡^	Ref (1.00)	0.31 (0.11–0.89)	0.15 (0.04–0.52)	0.002
**No**				
MIND values	< 19.39	19.39 to 22.23	> 22.23	
Median score	17.0	21.0	25.0	
cancer /control	34/63	19/63	28/63	
Crude	Ref (1.00)	0.57 (0.29–1.12)	0.84 (0.45–1.56)	0.575
Model 1^*^	Ref (1.00)	0.55 (0.28–1.08)	0.82 (0.44–1.54)	0.535
Model 2^†^	Ref (1.00)	0.54 (0.27–1.09)	0.84 (0.44–1.59)	0.574
Model 3^‡^	Ref (1.00)	0.50 (0.24–1.03)	0.81 (0.42–1.56)	0.513

^*^Model 1: adjusted for age and age at first pregnancy

^†^Model 2: adjusted for model 1 and family history of cancer (yes, no), menopausal status (yes, no), anti-inflammatory drugs (yes, no), and vitamin D supplement (yes, no)

^‡^Model 3: adjusted for model 2 and energy, BMI, physical activity, smoking status (yes, no), and SES (high, low)

## Discussion

To the best of our knowledge, there is limited evidence on the possible association between the MIND diet and BC risk. In the current study, we found that a higher MIND diet score is associated with decreased odds of BC; this strong inverse association between the MIND diet and BC risk is mainly observed in women with an abortion history.

To the best of our knowledge, evidence on the relationship between the MIND diet and the risk of BC is limited to a case-control study, which was conducted on newly diagnosed stage I-IV BC by Aghamohammadi et al. Aligned with our findings, their results showed that high adherence to MIND diet is related to reduced odds of BC when comparing the top tertile of the MIND diet score to the lowest tertile [20]. The advantage of our study over the previous study is that the history of abortion was considered in our analysis. Since the MIND dietary pattern is a hybrid version of DASH and Mediterranean diet compositions, individuals can benefit from the health effects of both diets in MIND form.

Considering that the MIND diet followed dietary characteristics similar to characteristics of two dietary patterns, including DASH and Mediterranean diets, the results of the current study can also indirectly support the result of previous studies that have reported the beneficial effect of the healthy dietary pattern, including DASH diet and Mediterranean diet in the prevention of BC development. It should be noted that the DASH diet has a protective effect against various types of cancer, including colorectal and breast cancers [28, 29]. A systematic review and meta-analysis of cohort studies have revealed that the DASH diet, as a healthy dietary approach, was related to a lower risk of different types of cancers such as endometrial, lung, hepatic, colon, rectal, colorectal, and breast cancer [30]. Also, Jones-McLean demonstrates the beneficial effect of the DASH diet on decreasing the risk of colorectal cancer [31]. Another study by Soltani et al. showed a negative relationship between DASH diet adherence and the odds of BC [32]. In line with previous findings, Mohseni et al. expressed that following the DASH diet decreases colorectal cancer risk [33]. Furthermore, Toorang et al. indicate a 30% reduction in BC risk following the DASH diet [34].

On the other hand, the Mediterranean diet is a kind of diet characterized by a high amount of whole grains, legumes, vegetables, fruit, nuts, extra-virgin olive oil, moderate consumption of fish, dairy and red wine, and a low intake of red or processed meats [35]. Due to the high anti-inflammatory and antioxidant properties of the Mediterranean diet, it can prevent or alleviate cancer progression by decreasing DNA impairments and cell proliferation [35, 36]. In this regard, Brandt and Schulpen confirm previous findings in a meta-analysis design study and claim that adherence to the Mediterranean diet decrease BC incidence, especially receptor-negative types [37]. Moreover, Castelló et al. express the benefits of the Mediterranean diet in reducing the risk of all subtypes of BC [38]. Also, two studies by Trichopoulou et al. and Turati et al. claim that higher compliance with the Mediterranean diet reflects lower BC incidence [39, 40]. However, Fung et al. documented no significant relationship between the risk of colorectal cancer and the Mediterranean diet in middle-aged women and men [41].

Our results suggest a potential inverse association between the MIND diet and BC in those with a history of abortion. In general, two reasons explained the inverse association of MIND diet scores with BC in those with abortion experiences. First, the completed pregnancy period protects against BC compared to women with abortion experiences. Second, women with induced abortions have high hormone levels in early pregnancies but without final differentiation of breast cells during late pregnancies. This can aggravate carcinogen generation [42].

On the other hand, there is considerable evidence about the impact of inflammation on abortion, and different dietary compounds can influence it. Mediterranean and DASH diets are two healthy dietary patterns associated with lower risks of inflammation [43–45]. In line with these findings, Ghosh Roy et al. revealed that abortions are predictors of BC [46], and as Vahid et al. showed, consumption of a pro-inflammatory diet increased the risk of abortion compared to those with an anti-inflammatory diet [43]. So, it is suggested that since women with a history of abortion are likely at higher risk for inflammation and breast cancer, the effect of the MIND diet may be more pronounced in these groups.

The MIND diet, as a plant-based diet, is a rich source of flavonoids, carotenoids, vitamin E, and folate due to its recommendation of whole grains, fruits, and vegetables, particularly berries and green leafy vegetables [47]. Since pro-inflammatory cytokines, reactive oxygen, and nitrogen species (RONS) activate Akt/PI3K/mTOR signaling and stimulate oncogenesis and tumor progression in BC [48, 49], the MIND diet might suppress these mechanisms through its antioxidant and anti-inflammatory properties.

The current study has several strengths that make the findings valuable. The first study assessed the role of the MIND diet in preventing BC risk in women based on their abortion history. Also, we have used valid and reliable questionnaires for assessing physical activity and dietary intake. Using multiple logistic regression models with adjusting various potential confounders is another strength of the current study. However, some limitations should be noted. Since our study is observational, selection and non-response bias are possible. On the other hand, we used FFQ as a retrospective tool for dietary assessment, which may cause recall bias.

In conclusion, our findings revealed that higher adherence to the MIND diet was associated with decreased BC odds, especially among women with a history of abortion. Further investigations are suggested to assess the possible protective role of the MIND diet in the development of BC prospectively in large cohort studies. Also, it is suggested to examine the effectiveness of this diet with a higher level of evidence by designing interventions based on the MIND diet.

## Data Availability

The datasets generated and analyzed during the current study are not publicly available due to our institute policies but are available from the corresponding author upon reasonable request.
